# Everolimus in pituitary tumor: a review of preclinical and clinical evidence

**DOI:** 10.3389/fendo.2024.1456922

**Published:** 2024-12-16

**Authors:** Zihong Yao, Hui Chen

**Affiliations:** ^1^ The Second Clinical Medical College of Lanzhou University, Lanzhou, Gansu, China; ^2^ Department of Endocrinology and Metabolism, Lanzhou University Second Hospital, Lanzhou, Gansu, China

**Keywords:** Everolimus, pituitary tumor, PI3K/AKT/mTOR, blood-brain barrier, safety

## Abstract

Although pituitary tumors (PTs) are mostly benign, some PTs are characterized by low surgical resection rates, high recurrence rates, and poor response to conventional treatments and profoundly affect patients’ quality of life. Everolimus (EVE) is the only FDA-approved mTOR inhibitor, which can be used for oral treatment. It effectively inhibits tumor cell proliferation and angiogenesis. It has been administered for various neuroendocrine tumors of the digestive tract, lungs, and pancreas. EVE not only suppresses the growth and proliferation of APT cells but also enhances their sensitivity to radiotherapy and chemotherapy. This review introduces the role of the PI3K/AKT/mTOR pathway in the development of APTs, comprehensively explores the current status of preclinical and clinical research of EVE in APTs, and discusses the blood-brain barrier permeability and safety of EVE.

## 1 Introduction

Pituitary tumors (PTs) are a group of tumors originating from the adenohypophysis, neurohypophysis, and remnants of the squamous epithelial cells of the embryonic craniopharyngeal duct, accounting for nearly 10%-25% of all intracranial tumors (1–4). Although PTs are mostly benign, some adenomas present with radiological signs of invasion and progress much faster than typical adenomas. Despite administering standard treatments, these tumors continue to grow and/or secrete excessive hormones and are categorized as aggressive pituitary tumors (APTs) (5–7). APTs account for 0.5%-10% of all PAs, with an incidence rate of approximately 0.1 to 0.2 cases per 100,000 individuals (8–11). They are characterized by aggressive invasion and high recurrence rates and significantly affect patients’ quality of life, which makes clinical management challenging (12–14).

Surgery is the primary treatment modality for all PTs except Prolactinoma (PRL-PAs). Although surgery can ameliorate the compressive effects of the tumor and partly reduce hormone secretion, the low rate of complete resection and high recurrence rates are often attributed to the widespread infiltration of critical structures, such as the sole of the saddle, slopes, or cavernous sinuses (15, 16). Reports indicated that 77% of APTs patients underwent at least two surgeries, with 28% requiring a minimum of four procedures (17). Radiotherapy is recommended for PTs with postoperative tumor growth and inadequate drug therapy; however, its effectiveness remains uncertain. The study showed that 45% of 143 patients had tumor shrinkage after radiotherapy, however nearly 40% received repeat radiotherapy after 5.4 years due to tumor progression (18). Radiotherapy may also lead to hypopituitarism, optic nerve damage, or cognitive deficits (19–22). The 2018 update of the European Society of Endocrinology guidelines on APTs and pituitary carcinomas recommended temozolomide (TMZ), an alkylating agent, as the first-line chemotherapeutic agent for APTs (23–27). However, one study indicated that only 9.6% of patients achieved complete remission with TMZ (18). Moreover, the long-term use of alkylating agents may increase the risk of malignancies, such as lymphomas and leukemias (28). Therefore, effective treatment strategies for APTs are currently lacking.

As a critical regulator of cellular growth (29–32), metabolism (32–34), and apoptosis (35–38), the mTOR pathway has recently received much attention in tumorigenesis (39–42). Everolimus (EVE), the only approved oral mTOR inhibitor can effectively suppress tumor cell proliferation, ameliorate cellular oxidative stress, and show anti-angiogenic properties (43–47). The Food and Drug Administration has approved EVE for treating neuroendocrine tumors (NETs) originating from the digestive tract, lungs, or pancreas (48–50). Overactivation of the mTOR pathway is also evident in PTs, a NETs (51, 52). Compared to normal pituitary tissue, significantly higher phosphorylation levels of nuclear p-AKT and cytoplasmic p-S6 and overall phosphorylation of eukaryotic translation initiation factor 4E-binding protein 1 (4EBP1) have been observed in PTs (53). The study demonstrated that EVE inhibited adenoma cell proliferation and concurrently decreased hormone secretion (54). Several large-scale clinical trials have shown that EVE has good overall tolerability and leads to a few severe adverse reactions (55–59). Therefore, EVE may serve as a potential alternative treatment option for patients with PTs who are resistant to conventional treatments. This article reviewed the research progress of EVE in PAs and other NETs, and investigated its blood-brain barrier (BBB) permeability and drug safety, providing new options for managing PTs.

## 2 Molecular mechanisms of function of EVE in PTs

### 2.1 Overview of the PI3K/AKT/mTOR pathway

**Figure 1:**
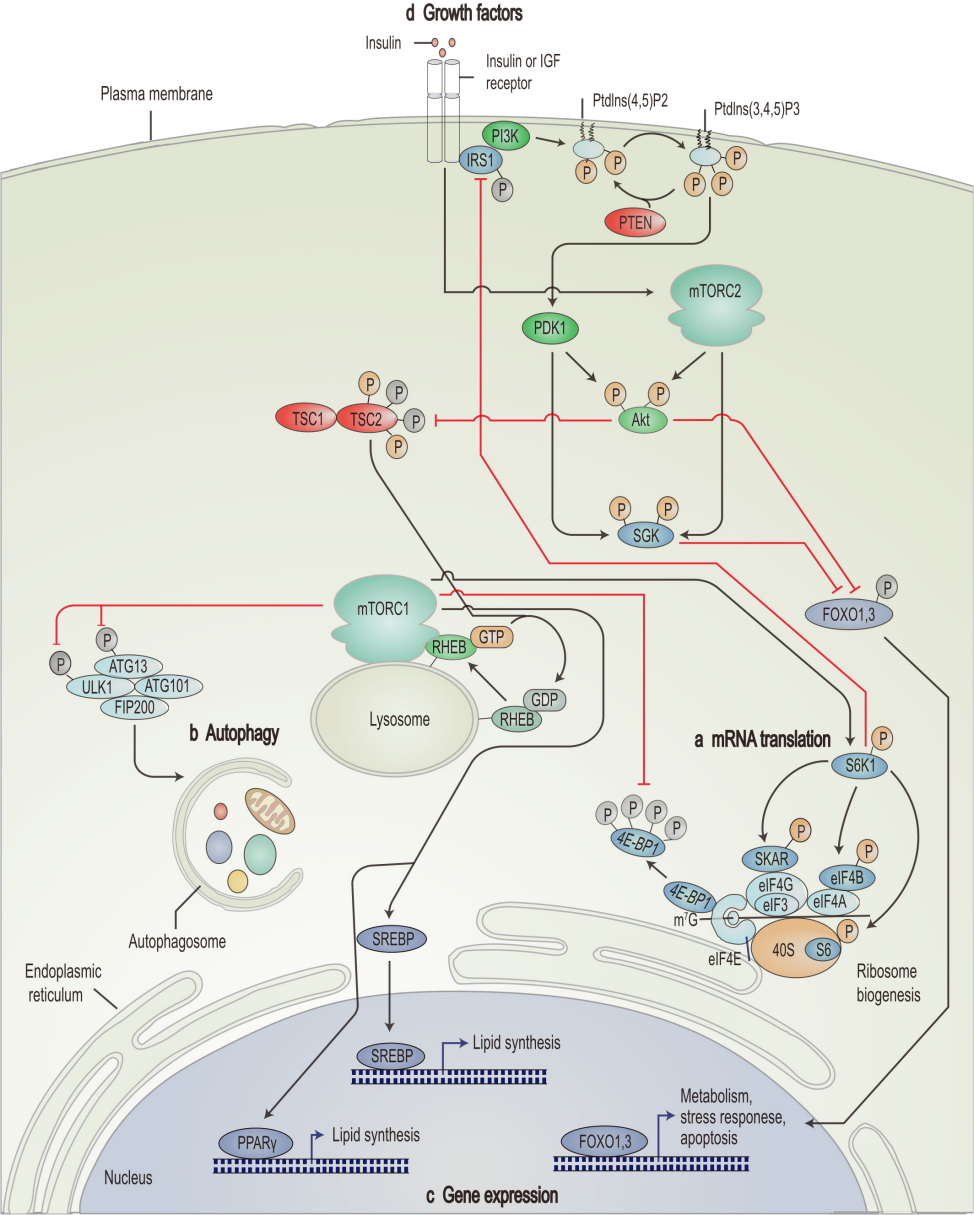
Schematic overview of the PI3K/AKT/mTOR signaling pathway. mTORC1 promotes mRNA translation **(A)**, inhibits autophagy **(B)**, promotes lipid synthesis-related genes and represses expression of apoptosis-related genes **(C)** through nutrient signals generated by growth factors such as insulin and insulin-like growth factor **(D)**. IGF, insulin-like growth factor; Ptdlns(4,5)P2, phosphatidylinositol-4,5-bisphosphate; Ptdlns(3,4,5)P3, triphosphoinositide; IRS1, insulin receptor substrate 1; SGK, serum- and glucocorticoid-regulated kinase; FOXO, forkhead box protein O; RHEB, Ras homolog enriched in brain; ATG, autophagy-related; ULK1, UNC51-like kinase 1; FIP200, 200 kDa FAK family kinase-interacting protein; eIF, eukaryotic translation initiation factor; 4E-BP1, eIF4E-binding protein 1; S6K1, ribosomal S6 kinase; SKAR, S6K1 Aly/REF-like target; SREBP, sterol regulatory element-binding protein; PPARγ, peroxisome proliferator-activated receptor-γ. [Adapted from Zoncu et al. (139)].

The PI3K/AKT/mTOR pathway plays a central role in signal transduction in organisms. It is involved in several biological processes, such as cellular metabolism, proliferation, and angiogenesis (60–62). It plays a crucial role in the development and progression of tumors (**Figure 1**) (63). PI3K consists of the regulatory subunit p85α and the catalytic subunit p110α, functioning as an intracellular phosphoinositide kinase (64). Activated PI3K converts phosphatidylinositol-4,5-bisphosphate (PIP2) into triphosphoinositide (PIP3) in the plasmalemma (65, 66). PIP3, a second messenger, recruits 3-phosphoinositide-dependent protein kinase 1 (PDK1), Akt, and serum- and glucocorticoid-regulated kinase to the plasma membrane (67–69).

Akt, a central element of the PI3K/AKT/mTOR pathway, can be completely activated dependent on two critical phosphorylation sites (70–73). The phosphorylation of Thr308 by PDK1 partly activates Akt, whereas mTORC2 phosphorylation of Ser473 fully activates Akt (74, 75). Once activated, Akt phosphorylates multiple downstream targets, such as tuberous sclerosis complex (TSC) 2 and forkhead box protein O, thereby directly or indirectly affecting cell growth and survival (76, 77).

mTOR is another crucial target of Akt. It is a highly conserved serine/threonine kinase (78), which forms two mTOR complexes, mTORC1 and mTORC2, by recruiting other proteins and active factors (79). Activation of mTORC1 relies on the phosphorylation of intracellular proteins, such as TSC (80). TSC possesses two subunits: TSC1 (hamartin) and TSC2 (tuberin) (81). Akt phosphorylates TSC2 and inhibits the negative regulatory effect of the TSC1-TSC2 complex on Ras homologue enriched in the brain, thereby activating mTORC1 (82). Once activated, mTORC1 also affects the phosphorylation of ribosomal S6 kinase (S6K1, p70S6k) and 4EBP1, further enhancing related gene expression and translation (**Figure 1A**). Besides, mTORC1 inhibits autophagy (**Figure 1B**), stimulates genes involved in lipogenesis (**Figure 1C**), and regulates apoptosis (**Figure 1D**), facilitating the rapid proliferation of cancer cells (83–86). mTORC1 effectively enhances angiogenesis by regulating hypoxia-inducible factor 1 alpha (87).

Phosphatase and tensin homologue (PTEN)-mediated hydrolysis of PIP3 to PIP2 and P70S6K-induced phosphorylation of insulin receptor substrate 1 (IRS1) negatively controls the PI3K/AKT/mTOR pathway (88, 89).

### 2.2 The involvement of the PI3K/AKT/mTOR pathway in the development and progression of PTs

Extensive research has been conducted on the alterations of the PI3K/AKT/mTOR pathway components in PTs (90). In a study involving 53 PAs, growth hormone-secreting pituitary adenomas (GH-PAs) (10/14, 71%) and non-functional pituitary adenomas (NFPAs) (11/33, 33%) showed a significant association with the mTOR pathway, compared to the control group (1/5, 20%) (91).

*PIK3CA* encodes the PI3K protein p110 subunit, which shows marked amplification and mutation in PTs. Murat et al. (92) found that 12.1% of PTs harbor *PIK3CA* mutations, whereas 21.2% of cases show gene amplification. Lin et al. (93) reported that *PIK3CA* mutations can be found in 9% of APTs, with 20%-40% of PTs displaying *PIK3CA* amplification. Another study showed that compared to normal pituitary tissue, the expression and phosphorylation levels of AKT and mTOR are elevated in PTs, whereas the expression level of PTEN is decreased (94).

The downstream effectors of mTOR, pS6, and eukaryotic translation initiation factor 4E (eIF4E) have been widely investigated in PTs. Compared to normal pituitary tissue, the pS6/eIF4E is more often activated in PTs (33%-71% vs. 20%) (95). In mice carrying mutations predisposing to PTs, the unregulated overexpression of p70S6k/S6 in PTs tissues compared to adjacent brain tissue (96). Dworakowska et al. (97) found no significant differences in the expression of p-mTOR, total mTOR, TSC2, and p70S6K between PTs and the control group; However, they did find the expression of c-MYC, a target of AKT and an oncogene, is elevated in PTs.

The mTOR pathway and its regulators are significantly linked to PTs invasion, staging, and tumor growth, and provide important predictive and prognostic value for PTs patients (98). Cai et al. (94) found that invasive PTs exhibit higher levels of AKT and lower levels of PTEN compared to non-invasive PTs. Further study of 95 PTs revealed that the expression of mTOR regulation-related proteins was negatively correlated with cavernous sinus invasion (98). Zhang et al. (99) discovered that lactate secreted by PTs cells promotes the polarization of tumor-associated macrophages via the mTORC2 signaling pathway, thereby enhancing the release of CCL17. This event promotes the invasion of PTs cells through the CCL17/CCR4/mTORC1 axis. It is worth noting that another study assessed the relationship between mTOR activity and PTs size, volume, Ki-67%, Knosp grade, and expression of somatostatin receptors, but found no significant correlations (95). This variability may be attributable to differences in sample size, experimental methods, and the heterogeneity of study subjects. To better understand the role of the mTOR pathway in PTs, larger studies encompassing various stages, degrees of invasion, and molecular subtypes are required.

Notably, there are variations in the PI3K/AKT/mTOR pathway activity among different subtypes of PTs. Analysis of 53 pituitary samples, including GH-PAs, NFPAs, and ACTH-secreting pituitary adenomas (ACTH-PAs), revealed elevated mTOR activity (estimated by the pS6/eIF4E ratio). GH-PAs exhibited the highest mTOR pathway activity, followed by NFPAs, while ACTH-PAs showed lower mTOR pathway activity (95). In the future, it is necessary to further explore the differences in mTOR pathway activity among various PTs subtypes, which is important for the development of individualized therapeutic regimens. Additionally, further studies on molecular mechanisms are needed to elucidate the factors contributing to the low mTOR pathway activity in ACTH-PAs.

### 2.3 The anti-tumor mechanisms of EVE

There is an intricate mechanism underlying the anti-tumor effects of EVE. EVE binds to FK506 binding protein 12 in mTORC1 and the FKBP-rapamycin binding domain, thereby inhibiting the activity of mTORC1 and its downstream molecules, blocking mRNA translation, leading to cell cycle arrest in the G1 phase, and enhancing tumor cell apoptosis (100). In addition, EVE induces the dissociation of raptor from mTORC1, preventing the phosphorylation of raptor by S6K1 and 4EBP1, thereby inhibiting protein synthesis and transcription (100). Moreover, EVE effectively induces the formation of autophagosomes in tumor cells, thereby increasing the LC3II/I and Beclin 1 expression, decreasing p62 expression, and potentiating autophagy (101). EVE inhibits tumor angiogenesis by directly targeting the mTOR in vascular endothelial cells, thereby suppressing their proliferation (102). Additionally, EVE decreases the synthesis of angiogenesis-promoting factors induced by hypoxia-inducible factors (102). Some studies have shown that in addition to the AKT-dependent pathway, EVE can activate mTOR through non-AKT pathways, such as Ras/MEK/ERK, thus exhibiting antitumor effects (103).

## 3 Current research status of EVE in PTs

After searching databases including pubmed, web of science, embase, and scopus, we included 12 studies from six countries on EVE treatment of PTs. These studies covered cellular, animal and human levels, with interventions including EVE monotherapy and combination therapies. Detailed data on EVE treatment for PTs are presented in **Table 1**. This paper provides a comprehensive review and discussion of previous studies across cellular, animal, and human levels.

**Table 1:** Overview of research on EVE therapy for pituitary tumors.

Research Group	Country	Research Level	Research Object	Intervention	Major Contributions & Findings
Gorshtein/2009 (104)	Israel	Cellular	GH3 cell lines	EVE	EVE inhibited the mTOR pathway and decreased the number of GH3 cells.
Zatelli/2010 (105)	Italy	Cellular, tissue	Human-derived NFPAs cells, 40 NFPAs tissues	EVE, IGF-1, SOM230	Evaluated the effects of EVE on mTOR pathway, cell viability, and apoptosis in NFPAs cells. EVE combined with SOM230 exhibited synergistic effects.
Sukumari-Ramesh/2011 (109)	USA	Cellular	GH3 cell lines, MMQ cell lines	EVE	EVE increased the sensitivity of PAs cell lines to radiotherapy.
Jouanneau/2012 (112)	France	Human body, tissue	1 ACTH adenocarcinoma, 17 ACTH-PAs, and their tissues	EVE+ Octreotide	The mTOR pathway was mildly activated in all ACTH-PAs. EVE monotherapy and combination therapies could not control tumor growth and ACTH secretion.
Donovan 2016 (113)	USA	Human body	1 refractory ACTH-PAs	EVE	The patient’s condition stabilized after using EVE. The patient finally died from systemic metastasis.
Jalali//2016 (111)	Canada	Animal	Mouse PAs model	EVE	Tumors formed by GH4C1 cell lines carrying different genotypes exhibited varying levels of mTOR pathway activation and growth rates. EVE reduced tumor volume, lowered GH levels, and inhibited p-S6.
Rubinfeld/2016 (107)	Israel	Cellular	MtT/E cell lines, human-derived NFPAs cells	EVE+Torin1	EVE combined with Torin1 inhibited cyclin D3 and p21 expression, and reduced Akt-Ser473 phosphorylation, with superior efficacy compared to monotherapy.
Pivonello/2018 (108)	Italy	Cellular	Rat GH3 cell lines, human-derived GH-PAs cells	EVE+PI3Ki	EVE combined with PI3Ki synergistically affected cell and colony survival. PI3Ki enhanced the effects of EVE by modulating the PI3K/Akt/mTOR and MAPK pathways.
Di Pasquale/2018 (110)	Italy	Cellular	GH3 cell lines, GH4C1 cell lines	EVE, IGF1	EVE inhibited IGF1-induced GH3 cell survival and GH secretion through the PI3K/Akt/mTOR pathway. EVE significantly reduced GH4C1 cell viability and increased p-Akt levels.
Zhang/2019 (53)	California	Human body, tissue, Cellular	1 refractory PRL-PAs, mice GH3 cell lines	EVE+CAB	Patient’s tumor shrank, and the blood levels of PRL decreased and remained stable for one year. EVE combined with CAB had an additive effect in suppressing PRL secretion but not on PAs cell proliferation.
Mangili/2022 (90)	Italy	Cellular	Human NFPAs cells, MMQ cell lines	EVE+CAB	Nearly 64% of cells exhibited resistance to EVE, which was reversed by 78% after adding CBA. The p-Akt/total Akt ratio was significantly increased in resistant cells.
Lin/2023 (54)	USA	Human body	4 refractory PRL-PAs	EVE	Three of the patients achieved biochemical remission. All patients experienced clinically significant benefits due to the inhibition of tumor growth.

PAs, Pituitary Adenoma; NFPAs, Non-Functional Pituitary Adenomas; ACTH-PAs, ACTH-Secreting Pituitary Adenoma; PRL-PAs, Prolactinoma; EVE, Everolimus; CAB, Cabergoline; IGF1, Insulin-like Growth Factor 1; PI3Ki, PI3K inhibitor; SOM230, Pasireotide; p70S6K, ribosomal S6 kinase; GH, Growth Hormone; ACTH, Adrenocorticotropic Hormone; PRL, Prolactin.

### 3.1 Cellular level

EVE has been extensively studied for its efficacy in treating PTs. Gorshtein et al. (104) first demonstrated that EVE inhibits the phosphorylation of p70S6K by blocking the mTOR pathway, thereby inducing G0/G1 cell cycle arrest and decreasing the survival of GH3 cells. Another study observed that EVE may inhibit IGF-1-induced GH3 cell survival and suppress GH secretion via the PI3K/Akt/mTOR pathway. Zatelli et al. (105) further support these findings. They applied different concentrations of EVE (1 nM-1 μM) to the primary cultures of human NFPAs, revealing that EVE inhibited p70S6K activity (-20%), reduced cell viability (70%), promoted apoptosis (+30%), and inhibited the proliferative and anti-apoptotic effects of IGF-1. Regazzo et al. (106) found that EVE can significantly decrease the survival of quiescent gonadotroph adenoma cells, further validating its potential for widespread application in different subtypes of PTs.

To improve the efficacy of EVE monotherapy and mitigate resistance, researchers have started exploring its combination with additional drugs or therapeutic strategies. Previous studies have indicated that the combination of EVE with Torin1 can markedly decrease the viability of MtT/E pituitary cells and human-derived NFPAs cells and the expression of cyclin D3 and p21, surpassing the effects of single-agent therapy (107). Compared to EVE monotherapy, EVE combined with pasireotide yielded similar cumulative effects in NFPAs cells (105). EVE combined with PI3K inhibitors (PI3Ki) exerted synergistic effects on cell and colony survival in rat GH3 and human GH-PAs cell lines, which may be attributed to the fact that PI3Ki enhanced the anti-tumor effect of EVE by modulating the PI3K/Akt/mTOR and MAPK pathways (108). However, some studies indicate that EVE combined with cabergoline (CAB) showed only additive effects in inhibiting PRL secretion, without significant synergistic effects on tumor cell proliferation (53). Moreover, EVE had been shown to enhance the sensitivity of GH3 cell lines to radiotherapy (109). These studies offer new insights into EVE combination therapy for treating PTs, promising more effective and personalized options. However, despite its significant anti-tumor effects, the therapy’s effectiveness depends on factors like drugs choice, cell line specificity, and treatment stage. Future research should explore optimal combinations, focusing on the mechanisms of combined therapy and validating their feasibility and efficacy through clinical trials.

Recent studies have revealed key findings regarding the role of EVE combination therapy in overcoming PTs resistance. Studies have shown that nearly 64% of primary cultured NFPAs cells are resistant to EVE (90), often associated with a significant increase in p-AKT (110) and p-AKT/total-AKT ratio (90). Researchers have tried EVE in combination with drugs like CAB (90) or Torin1 (107), which has proven effective in attenuating EVE-induced Akt-Ser473 phosphorylation levels and reversing approximately 78% of the resistance (90). However, another study presented an alternative view, showing that EVE combined with Gleevec may further activate p-AKT and exacerbate drug resistance. These findings highlight the complexity and challenges in selecting combination therapy strategies, suggesting that the treatments effectiveness may vary with patient populations or specific drug combinations. Further studies on molecular mechanisms are needed to clarify the underlying biological basis.

### 3.2 Animal level

There are four main types of animal models used in PTs research: cell line-derived xenografts (CDX), patient-derived xenografts (PDX), environmentally induced models, and genetically engineered mouse (GEM) models (63). Although CDX and *in vitro* cultured animal-derived cell lines are commonly used in pituitary research, their limitations, including the loss of genetic heterogeneity in CDX and differences from the human tumor microenvironment, limit their suitability for long-term studies. Conversely, the use of PDX models is relatively limited. Currently, there are no commercially available human PTs cell lines, making the cultivation of primary human-derived PTs cells challenging and limited to short-term studies. Additionally, while rodent cell lines are more accessible, their pathophysiological characteristics differ from those of humans, posing challenges for developing models that accurately reflect human biology. Emerging techniques, like pituitary induction using human induced pluripotent stem cells, show promise for creating reliable human PTs models. These methods aim to cultivate cell lines with human PTs characteristics *in vitro*, potentially overcoming current model limitations. Future exploration of these approaches may significantly advance PTs diagnosis and treatment.

Jalali et al. (111) established xenograft mouse models employing GH4C1 cell lines with different FGFR4 genotypes (wild-type G388, polymorphic R388, and parental controls) to assess tumor growth rates and Ki-67 expression. They then analyzed the mTOR pathway activation (p-S6 and p-4EBP1) and administered EVE to assess its effect on tumor growth. They observed that tumors with G388 and R388 genotypes grew faster than parental controls, and showed increased Ki-67 expression. These findings suggest that the FGFR4 genotype may accelerate tumor growth by enhancing cell proliferation. Further research revealed significantly increased p-S6 and p-4EBP1 activity in tumors with G388 and R388 genotypes. EVE treatment reduced p-S6 levels across all genotypes, suggesting that EVE may effectively inhibit tumor growth by suppressing the mTOR pathway (111). This study uses animal models to explore the impact of FGFR4 genotypes on PTs growth rates and the involvement of the mTOR pathway, while also assessing EVE’s potential in slowing intracranial tumor growth. The findings improve understanding of PTs biology and support the development of new treatment strategies. Given the effect of PTs genotypes on EVE efficacy, exploring personalized EVE treatments for different genotypes could be highly significant.

### 3.3 Human level

Previous clinical studies on EVE treatment for PTs have mainly focused on patients with APTs, with most literature being case reports. Several studies suggest that EVE partially inhibits APTs growth and improves clinical symptoms. Zhang et al. (53) reported a patient with PRL-PAs who were resistant to standard treatments, such as CAB. The addition of EVE to CAB markedly decreased PRL levels and tumor regression. Additionally, Lin et al. (54) described four patients with aggressive PRL-PAs, three of whom finally achieved sustained stabilization of their disease and their PRL levels decreased after adding EVE to CAB therapy. Of the four patients, two discontinued TMZ and pasireotide before adding EVE (patients 2 and 3). Jouanneau et al. (112) found that neither EVE monotherapy nor its combination with octreotide can effectively reduce tumor growth or ACTH secretion. This may be attributed to the weak activation of the mTOR pathway in ACTH-PAs. However, Donovan et al. (113) found that patients with ACTH-PAs harboring STK11 mutations respond better to EVE. This suggests that when identifying patients who may be suitable for treatment with EVE, it is essential to consider not only the mTOR activity of PTs but also other factors that may affect patients’ sensitivity to EVE, such as mutations of specific genes, levels of biological markers (114), and the degree of tumor differentiation (115).

In summary, although EVE has demonstrated potential therapeutic effects in the preclinical studies of PTs, data on its efficacy as a treatment in the clinical management of PTs remain limited. The exact efficacy of EVE in clinical practice is still unclear and it is not known whether EVE should be added to ineffective first-line treatment options (e.g.CAB, pasireotide, etc.) or whether current first-line treatments should be discontinued before initiating EVE monotherapy or combination therapy. Future prospective, multicenter clinical trials are needed to explore these issues.

## 4 Molecular mechanisms of EVE in Other NETs

Given the limited data on EVE in PAs, we elucidated its mechanism of action by investigating its effects in other NETs. The PI3K/Akt/mTOR pathway plays a crucial role in the development of NETs. French researchers Boilève et al. (116) conducted a retrospective analysis of real-world data on precision treatment for patients with NETs, revealing that the mTOR pathway is the most frequently altered pathway in these patients (24%). Downregulation of the PTEN and TSC2 genes in the PI3K/Akt/mTOR pathway is significantly associated with shorter disease-free survival and overall survival in patients with NETs (117).

EVE exerts its anti-NET effects through several mechanisms. Firstly, EVE exerts an inhibitory effect on the proliferation of NET cells (118, 119). *In vitro* and *in vivo* experiments, EVE, either alone or in combination with cytotoxic agents, can block the growth and proliferation of NETs cells, primarily by inhibiting protein synthesis (120, 121). Zitzmann et al. (118) treated BON cells, a human cell line of pancreatic NETs with constitutive activation of the PI3K/AKT/mTOR signaling pathway, with various concentrations of EVE and found that it inhibited the growth of BON cells in a dose-dependent manner. Grozinsky-Glasberg et al. (119) investigated the effects of octreotide, EVE, and their combination on cell proliferation and kinase activation in NET cell lines (the rat insulinoma cell line, INS1), finding that both octreotide and EVE inhibited cell proliferation and reduced the phosphorylation of Akt downstream targets (TSC2, mTOR, and p70S6K).

Secondly, EVE exerts anti-angiogenic effects on NETs. NETs are among the most vascularized tumors discovered to date. In the phase III RADIANT-3 clinical trial, Yao et al. (122) analyzed the levels of vascular endothelial growth factor (VEGF), platelet-derived growth factor (PGF), basic fibroblast growth factor, soluble vascular endothelial growth factor receptor-1 (sVEGFR-1), and soluble vascular endothelial growth factor receptor-2 (sVEGFR-2) in patients before and after treatment with EVE. They found that EVE significantly reduced the levels of sVEGFR-2 and PGF compared to placebo. Furthermore, EVE, in combination with somatostatin analogs, demonstrated a synergistic anti-angiogenic effect, whereby EVE reduced the production of VEGF by tumor cells by inhibiting the mTOR-HIF-1α pathway, while somatostatin analogs acted directly or indirectly on stromal endothelial cells and monocytes expressing somatostatin receptors (123).

Additionally, EVE induced cell cycle arrest and apoptosis in NETs while promoting autophagy. EVE reduced the phosphorylation levels of downstream targets of the PI3K/AKT pathway, including TSC2, mTOR, and p70S6K, leading to cell cycle arrest in the G0/G1 phase and inducing apoptosis (118). Furthermore, EVE affects autophagy by downregulating the AKT signaling pathway. mTORC1 inhibits autophagosome formation and initiation of autophagy by phosphorylating UNC51-like kinase 1 (ULK1); however, the mTOR inhibitor EVE can suppress mTORC1 and prevent the phosphorylation of ULK1, thereby accelerating autophagy (124). Histopathological studies indicate that the combination of chloroquine and EVE can significantly inhibit mTOR activity and the growth of NETs, while simultaneously suppressing the accumulation of autophagosomes and increasing apoptosis (124).

In summary, EVE exhibits multiple mechanisms of action in NETs, including inhibition of cell proliferation and angiogenesis, induction of cell cycle arrest and apoptosis, and promotion of autophagy. Future studies should investigate the combinatorial effects of EVE with other therapeutic modalities to improve treatment outcomes for patients with NETs.

## 5 Blood-brain barrier permeability of EVE

Chemotherapeutic agents’ permeability through the BBB directly affects their efficacy. EVE, a lipophilic compound, shows considerable BBB permeability and promising therapeutic effects against central nervous system tumors (125). EVE can modulate efflux mechanisms mediated by breast cancer resistance protein (BCRP) and P-glycoprotein (P-gp), improving its passage through BBB (126–128). Researchers have developed several drug delivery systems, such as osmotic pumps (129), convection-enhanced delivery (130), and interstitial therapies (131), to implant EVE directly into the tumor tissue or surrounding stroma, effectively bypassing the BBB. However, the effectiveness of these methods can be affected by factors in the tumor microenvironment, such as the extracellular matrix and brain’s lymphatic drainage system, which may restrict drug distribution and retention. To address this challenge, Han et al. (132) optimized drug distribution and retention in brain tumors using liposomal formulations. They developed liposomes with varying surface charges, PEGylation, and transition temperatures, and evaluated them for *in vitro* cellular uptake, distribution, and persistence in brain tissue. The study found that PEGylated liposomes with positive charges and high transition temperatures, especially EVE liposomes, showed significantly improved interstitial therapeutic efficacy for intracranial tumors. Future studies should focus on elucidating the mechanisms underlying EVE passage through BBB to optimize drug delivery strategies and enhance its clinical application in intracranial tumors.

## 6 Safety of EVE

Stomatitis, hyperlipidemia, and hyperglycemia are the most common adverse events associated with EVE (incidence rate ≥1/10), whereas severe adverse events (≥grade 3) are relatively rare (133). The incidence of adverse events associated with EVE is closely correlated with its blood concentration. In one study, when the peak concentration (C_min_) of EVE was <7.8 ng/mL and ≥7.8 ng/mL, 9%, and 14%-19% of patients experienced adverse reactions (134), respectively. Another study reported that for each two-fold increase in the C_min_ of EVE, the risk of severe adverse events increased by 1.5, whereas reducing the dose of EVE allowed patients to recover (135). A meta-analysis indicated that the risk of pulmonary adverse events significantly increases when the C_min_ of EVE exceeds 30 ng/mL (136). The blood concentration of EVE is considerably higher in patients experiencing dose-limiting toxicities (DLTs) than in those without toxicities (137). The cumulative incidence of DLTs significantly increased (HR: 4.87, 95% CI: 1.53-15.5) (138). There are significant variations in pharmacokinetic parameters of EVE among different individuals, such as clearance rates (range: 5.1-21.3 L/h/70 kg, coefficient of variation: 38.5%) and central distribution volume (range: 9.9-103.6 L/70 kg, coefficient of variation: 57.8%) (137). Therefore, blood drug concentrations should be monitored in patients receiving EVE treatment to ensure that their C_min_ remains within a safe range, enabling the early detection of adverse events and appropriate medical management.

## 7 Conclusion and prospect

The PI3K/AKT/mTOR pathway is overactivated in PTs. EVE, a selective kinase inhibitor, directly and durably inhibits mTOR, demonstrating vigorous anti-PTs effects. Previous studies have validated the therapeutic efficacy of EVE against PTs across cellular, animal, and human-level.

Although EVE shows potential in treating PTs, several issues remain. First, while low mTOR pathway activity has been observed in ACTH-PAs, the precise molecular mechanisms are unclear. Further research is required to identify specific mutations or regulatory mechanisms within this pathway in ACTH-PAs and their impact on EVE’s efficacy. Additionally, since EVE was recently approved, existing studies mainly address short-term outcomes and lack long-term follow-up data. Future efforts should involve large-scale, multi-center studies to evaluate EVE’s long-term efficacy and safety. Furthermore, no definitive biomarkers for treatment-resistant PTs have been identified, complicating early detection of APTs. Research should focus on discovering early predictive biomarkers and treatment response indicators for resistant PTs, as well as defining optimal treatment duration, sequencing, and combinations for personalized strategies. Given the rarity and heterogeneity of APTs, clinical management experience has accumulated slowly. Establishing national and international registries could improve understanding and management of APTs in the future.

## Glossary

ACTH: Adrenocorticotropic hormone
ACTH-PAs: ACTH-secreting pituitary adenoma
ATG: Autophagy-related
APTs: Aggressive Pituitary Tumors
BBB: Blood-Brain Barrier
BCRP: Breast Cancer Resistance Protein
CAB: Cabergoline
CDX: Cell line-Derived Xenografts
C_min_: Peak concentration
DLTs: Dose-limiting toxicities
eIF: Eukaryotic translation initiation factor
EVE: Everolimus
FDA: Food and Drug Administration
FIP200: 200 kDa FAK family kinase-interacting protein
FOXO: Forkhead box protein O
FPAs: Functional pituitary adenomas
GEM: Genetically Engineered Mouse
GH: Growth Hormone
GH-Pas: Growth Hormone-Secreting Pituitary Adenomas
IGF1: Insulin-like growth factor 1
IRS1: Insulin receptor substrate 1
NETs: Neuroendocrine Tumors
NFPAs: Non-functional pituitary adenomas
PTs: Pituitary Tumors
PDK1: 3-phosphoinositide-dependent protein kinase 1
PDX: Patient-Derived Xenografts
PGF: Platelet-derived Growth Factor
P-gp: P-glycoprotein
PI3Ki: PI3K inhibitor
PIP2: Phosphatidylinositol-4,5-bisphosphate
PIP3: Triphosphoinositide
PPARγ: Peroxisome proliferator-activated receptor-γ
PRL: Prolactin
PRL-Pas: Prolactinomas
PTEN: Phosphatase and tensin homologue
RHEB: Ras homologue enriched in the brain
S6K1/p70S6K: Ribosomal S6 kinase
SKAR: S6K1 Aly/REF-like target
SOM230: Pasireotide
SREBP: Sterol regulatory element-binding protein
sVEGFR-1: soluble Vascular Endothelial Growth Factor Receptor-1
sVEGFR-2: soluble Vascular Endothelial Growth Factor Receptor-2
TMZ: Temozolomide
TSC: Tuberous Sclerosis Complex
4EBP1: Eukaryotic translation initiation factor 4E-binding protein 1
ULK1: UNC51-like kinase 1
VEGF: Vascular Endothelial Growth Factor
